# Efficacy and Safety of Gegen Qinlian Decoction for Pediatric Diarrhea: A Systematic Review and Meta-Analysis

**DOI:** 10.1155/2022/4887259

**Published:** 2022-09-08

**Authors:** Dan Wang, Chao-Ran Bi, Hai-Yan Jiang, Yi-Jing Li, Wen-Ping Zhang, Yuan Liu, Yan-Jing Liu

**Affiliations:** ^1^Department of Endocrinology, Metabolism and Gastroenterology, Third Affiliated Clinical Hospital to Changchun University of Chinese Medicine, Changchun 130117, Jilin, China; ^2^College of Traditional Chinese Medicine, Changchun University of Chinese Medicine, Changchun 130117, Jilin, China; ^3^Department of Cardiology, Third Affiliated Clinical Hospital to Changchun University of Chinese Medicine, Changchun 130117, Jilin, China

## Abstract

**Objective:**

To evaluate the clinical efficacy and safety of Gegen Qinlian decoction in the treatment of pediatric diarrhea.

**Methods:**

A search for relevant RCTs was performed from which a systematic review and meta-analysis was conducted. This meta-analysis was registered at INPLASY (reference number ID: INPLASY202180105).

**Results:**

(1) Eleven trials involving 1126 patients were included in the meta-analysis. (2) Two trials recorded the adverse events. (3) The meta-analysis showed that compared with the control group, the experimental group has a significantly shorter duration of diarrhea in children (MD = −18.64, 95% CI (−23.76, −13.52), *P* < 0.00001), duration of fever (MD = −19.43, 95% CI (−25.76, −13.11), *P* < 0.00001), duration of vomiting [MD = −22.51, 95% CI (−29.92, −15.09), *P* < 0.00001], duration of correcting dehydration (MD = −23.35, 95% CI (−35.48, −11.22), *P*=0.0002), and the effective rate (OR = 4.64, 95% CI (3.12, 6.90), *P* < 0.00001).

**Conclusion:**

There were significant differences in the clinical efficacy in the treatment of pediatric diarrhea between the experimental and control groups. Thus, Gegen Qinlian decoction may have certain advantages in the treatment of pediatric diarrhea. In addition, we conclude the following: (1) the application of Gegen Qinlian decoction to treat this disease is recommended for >5 days. (2) We recommend conducting multicenter RCTs to avoid the impact of regional differences on the results. (3) We recommend using the unmodified Gegen Qinlian decoction, which may have better efficacy.

## 1. Introduction

Pediatric diarrhea is a digestive disorder in children caused by a variety of agents [1]. The main clinical symptoms are frequent and watery diarrhea accompanied by fever, vomiting, and abdominal pain. Dehydration and acid-base imbalance can also occur in severe cases [2]. In addition, long-term chronic diarrhea is often accompanied by complications, such as malnutrition, anemia, immune depression, and growth retardation [3]. Diarrhea is a common and frequently occurring disease in children. Some studies have reported that the average frequency of diarrhea in children <5 years is 3.2 times per year, and 10% of children 1–59 months die from diarrhea [4]. Studies have shown that diarrhea is the 5th leading cause of death in children <5 years [5].

Viral and bacterial infections are important causes of pediatric diarrhea. With respect to bacterial infections causing pediatric diarrhea, the spectrum of pathogens in poor areas of China is similar to Africa and southern Asia with a high detection rate of *Shigella*. Economically-developed regions have a spectrum of bacterial pathogens causing pediatric diarrhea similar to European and American countries, with *Escherichia coli*, *Salmonella*, and *Yersinia* most common [6]. With respect to viral infections, rotavirus is the primary virus that causes diarrhea in children in China and abroad [5, 7, 8]. Pediatric diarrhea has two peak seasons each year. One peak is from June to August, and the main pathogens are diarrhea-causing *Escherichia coli* and *Shigella*. The other peak is from October to December, and the main pathogen is the rotavirus [9]. In addition, the persistence of pediatric diarrhea is often directly related to juvenile diabetes and pneumonia [10]. Studies have shown that some herbal ingredients also have a good therapeutic effect on diabetic diarrhea and pneumonia [11, 12]. Thus, medicinal plants and herbal products could be a good source of antidiabetic medications [13, 14].

At present, most international guidelines recommend the application of oral rehydration salts for treatment. In the case of bacterial infections, antibiotics are also recommended [15–17]; however, the irrational use of antibiotics often leads to an imbalance in the intestinal flora and antibiotic resistance in children [18, 19]. Antimicrobial resistance is a major cause of clinical antimicrobial therapeutic failure [20]. Thus, the efficacy and safety of this treatment method is not fully accepted by people. Based on the results of some RCTs, if the Gegen Qinlian decoction (GQD) is combined with this treatment method, the efficacy and safety may be improved [21, 22]; however, no systematic reviews and meta-analyses have been published involving these RCTs. GQD is a classic Chinese medicine that was first recorded in Shang-Han-Lun of the Han Dynasty (202 BC-220 AD). GQD consists of Radix *Puerariae lobatae* (Ge Gen), Radix *Scutellariae* (Huang Qin), *Rhizoma coptidis* (Huang Lian), and Radix *Glycyrrhizae* (Gan Cao). This composition can dissipate the sickness on the surface and clean up the damp heat inside. It has been reported that GQD has significant antiviral, antibacterial, antipyretic, and gastrointestinal peristalsis inhibitory effects [23]. Therefore, GQD is widely used in the treatment of diarrheal diseases [24, 25].

Nevertheless, there are different opinions regarding the efficacy and safety of GQD in the treatment of pediatric diarrhea. Therefore, this study searched the relevant randomized controlled trials (RCTs). After the literature search, a comprehensive study was carried out using the meta-analysis method to evaluate the efficacy and safety of GQD in the treatment of pediatric diarrhea.

## 2. Methods

This article followed the requirements suggested by Cochrane Handbook [26], and our study was conducted following the Preferred Reporting Items for Systematic Reviews and Meta-analyses (PRISMA) statement [27], and the PRISMA Checklist (Table S1) can be found in the supplementary material. This meta-analysis was registered at INPLASY [28] (reference number ID: INPLASY202180105).

### 2.1. Search Strategy

Two researchers searched the literature in the China National Knowledge Infrastructure (CNKI; https://www.cnki.net), VIP database (https://www.cqvip.com), Wanfang database (https://www.wanfangdata.com.cn/index.html), China Biomedical Database (CBM; https://www.sinomed.ac.cn), and Pubmed database (https://pubmed.ncbi.nlm.nih.gov) according to the inclusion and exclusion criteria.

The following terms were used to search the title, subject, and key words for relevant RCTs: “Gegen Qinlian decoction;” “children's diarrhea;” “diarrhea in children;” “pediatric diarrhea;” “infantile diarrhea;” “children's enteritis;” “enteritis in children;” and “pediatric enteritis.” If necessary, the search included full text articles. The Search Strategy (Table S2) can be found in the supplementary material.

### 2.2. Inclusion Criteria

1. Research objects: the literature published in domestic and international journals or conference papers related to GQD in the treatment of pediatric diarrhea.
2. Literature type: the article must be an RCT.
3. Treatment method: the control group was treated with conventional comprehensive treatment or combined with other treatments other than GQD. The treatment group was treated with GQD alone or GQD was added to the treatment plan of the control group.
4. Research indicators (at least one): duration of diarrhea; duration of fever; duration of vomiting; time to correct dehydration; effective rate; and adverse events.

### 2.3. Exclusion Criteria

1. Does not meet the inclusion criteria.
2. RCT with incomplete data collection, inappropriate trial design, and inaccurate statistical methods.
3. Duplicate trials or data.
4. Experience summary, review, and case report;
5. Cell or animal experiments.

### 2.4. Study Selection and Data Extraction

According to the inclusion and exclusion criteria, the two researchers cross-checked and consulted experts to assist in the decision involving cases in which there was disagreement. Then, the two researchers extracted and summarized the author, year, sample size, average age, intervention measures, course of treatment, outcome indicators, adverse reactions, and other contents that were finally included in the trials. A table with the basic information of the included RCTs was created.

### 2.5. Quality Assessment

The literature quality assessment of this study was conducted using the risk of bias table recommended by the Cochrane Collaboration [26]. The table includes seven items: whether the randomized plan is clear; whether to hide the assignment; whether to blind during the test; whether to blind in the result analysis; whether the outcome data is complete; whether the results are selectively reported; and other sources of bias. The evaluation criteria are described as “yes” (low risk), “no” (high risk), or “unclear” (unclear). In the event of a disagreement during the evaluation process, the two researchers discuss the case first, and if the conflict cannot be resolved, the third researcher assists in the decision.

### 2.6. Data Analysis

Review Manager 5.4 software was used for statistical analysis. For continuous variables, the mean difference (MD) was used for statistical analysis, for dichotomous variables, the odds ratio (OR) was used, and the study confidence interval was set to 95%. Heterogeneity analysis was performed using the *I*^2^ test [29]. When *I*^2^ ≤ 50% or *P* ≥ 0.05 indicated no statistical heterogeneity, the fixed-effects model was used. Otherwise, a random-effects model was used [30]. The pediatric diarrhea curative effect of the treatment and control groups were compared using a forest diagram.

Sensitivity (one-by-one excluding the RCT method) and subgroup analyses were used to clarify the source of heterogeneity. A funnel plot was used to identify potential publication bias.

## 3. Results

### 3.1. Literature Search

**Figure 1:**
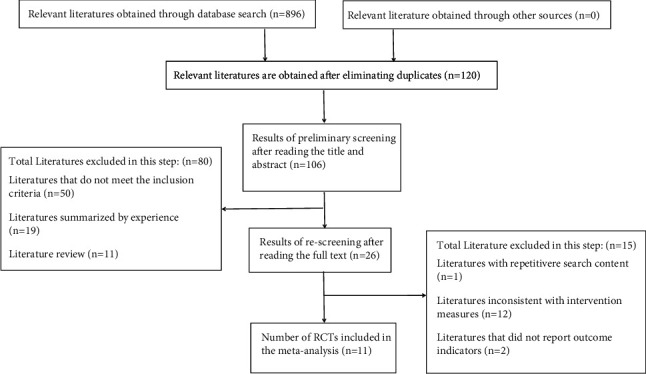
Flow diagram of RCT selection.

A total of 896 related articles were searched. By reading the title, abstract, and keywords, and according to the exclusion criteria, the articles that did not meet the standards were excluded. After rescreening, 11 RCTs [21, 22, 31–39] were included. Among the included RCTs, there were six in the past 5 years. The age of the participating children was 1-2 years. The disease course was typically 1–7 days in length. Montmorillonite powder and ribavirin are drugs commonly used to treat pediatric diarrhea. The main outcome indicators were duration of diarrhea, fever, vomiting, and dehydration. There were more RCTs conducted in southern than northern China. The main causes of disease were rotavirus and other viruses. The characteristics of the included RCTs are shown in Table 1. The RCT selection process is shown in Figure 1. The specific ingredients of GQD used in the 11 RCTs are shown in Table 2. As summarized in Table 2, modified GQD was more frequently used than GQD, but GQD was used in RCTs more frequently in the past 3 years. The medication is administered orally for 3–7 days. The main ingredients are *Puerariae lobatae* Radix (Ge Gen), *Scutellariae* Radix (Huang Qin), *Coptidis Rhizoma* (Huang Lian), and *Glycyrrhizae* Radix (Gan Cao).

### 3.2. Methodologic Quality Assessment

**Figure 2:**
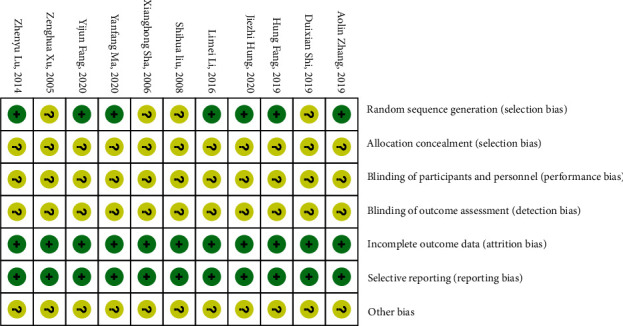
The risk of bias summary graph for the 11 included studies.

(1) Random sequence generation: four studies [21, 22, 32, 35] only mentioned the word, “random,” and seven studies [31, 33, 34, 36–39] described specific random methods. (2) Allocation concealment: none of the 11 studies [21, 22, 31–39] are described. (3) Blinding of participants and personnel: none of the 11 studies [21, 22, 31–39] are described. (4) Blinding of outcome assessment: none of the 11 studies [21, 22, 31–39] are described. (5) Incomplete outcome data: the outcome data of the 11 studies [21, 22, 31–39] are complete. (6) Selective reporting: the 11 studies [21, 22, 31–39] were fully reported. (7) Other bias: none of the 11 studies [21, 22, 31–39] are described (Figure 2).

### 3.3. Outcomes

#### 3.3.1. Duration of Diarrhea

**Figure 3:**
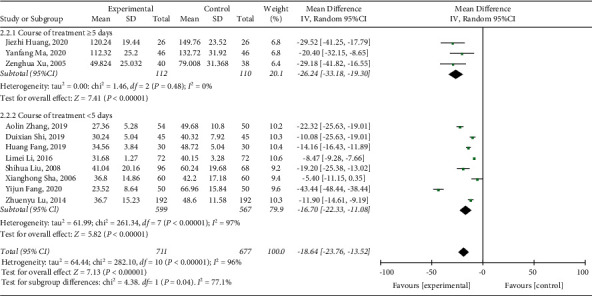
Forest plot and meta-analysis of the diarrhea duration.

Among the included studies, 11 [21, 22, 31–39] reported diarrhea duration. As shown in the forest plot (*I*^2^ = 96%, *P* < 0.00001), there was high heterogeneity. Therefore, a subgroup analysis was implemented. We reasoned that the source of heterogeneity was related to the course of treatment. The meta-analysis results were as follows: (MD = −18.64, 95% CI (−23.76, −13.52), *P* < 0.00001), suggesting that the diarrhea duration in the treatment group was lower than that in the control group (Figure 3).

#### 3.3.2. Duration of Fever

**Figure 4:**
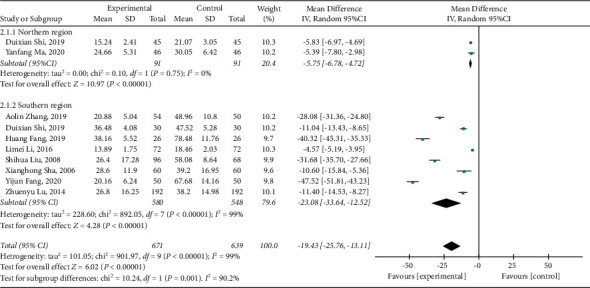
Forest plot and meta-analysis of the fever duration.

Among the included studies, 10 studies [22, 31–39] reported fever duration. As shown in the forest plot (*I*^2^ = 99%, *P* < 0.00001), there was high heterogeneity. Therefore, subgroup analysis was implemented. We reasoned that the source of heterogeneity was related to the regional difference. The meta-analysis results were as follows: (MD = −19.43, 95% CI (−25.76, −13.11), *P* < 0.00001), suggesting that the fever duration in the treatment group was lower than that in the control group (Figure 4).

#### 3.3.3. Duration of Vomiting

**Figure 5:**
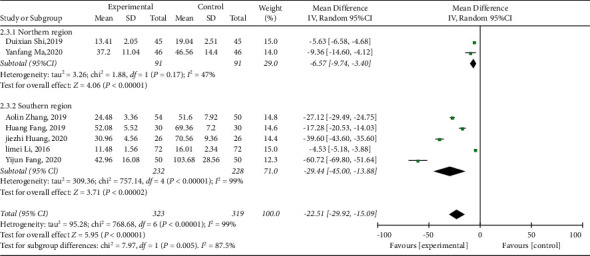
Forest plot and meta-analysis of the vomiting duration.

Among the included studies, seven [31–33, 36–39] reported the vomiting duration. As shown in the forest plot (*I*^2^ = 99%, *P* < 0.00001), there was high heterogeneity. Therefore, subgroup analysis was implemented. We reasoned that the source of heterogeneity was related to the regional difference. The meta-analysis results are as follows: (MD = −22.51, 95% CI (−29.92, −15.09), *P* < 0.00001), suggesting that the vomiting duration in the treatment group was lower than that in the control group (Figure 5).

#### 3.3.4. Duration of Correcting Dehydration

**Figure 6:**
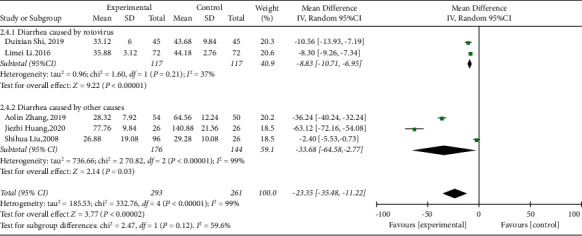
Forest plot and meta-analysis of the time to correct dehydration.

Among the included studies, 5 [31, 32, 35–37] reported the duration to correct dehydration. As shown in the forest plot (*I*^2^ = 99%, *P* < 0.00001), there was high heterogeneity. Therefore, subgroup analysis was implemented. We reasoned that the source of heterogeneity was related to the cause of the disease. The meta-analysis results were as follows: (MD = −23.35, 95% CI (−35.48, −11.22), *P*=0.0002), suggesting that the time elapsed to correct dehydration in the treatment group was less than that in the control group (Figure 6).

#### 3.3.5. Effective Rate

**Figure 7:**
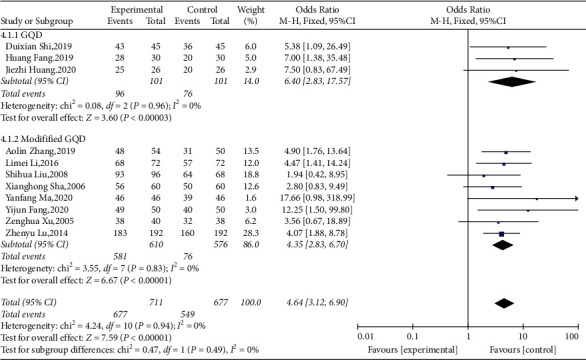
Forest plot and meta-analysis of the total effective rate.

Among the included studies, 11 [21, 22, 31–39] reported the effective rate. As shown in the forest plot (*I*^2^ = 0%,  *P*=0.94), there was no heterogeneity. Therefore, the fixed-effects model was used for the meta-analysis. We conducted subgroup analysis based on the modification status of Chinese medicine. The meta-analysis results were as follows: (OR = 4.64, 95% CI (3.12, 6.90), *P* < 0.00001), suggesting that the effective rate of the treatment group was greater than that of the control group. At the same time, the meta-analysis showed that the effective rate of TCM with GQD (OR = 6.40, 95% CI (2.33, 17.57), *P*=0.0003) was greater than modified GQD [OR = 4.35, 95% CI (2.83, 6.70), *P* < 0.00001] (Figure 7).

#### 3.3.6. Adverse Events

It has been reported that the incidence of adverse reactions in the treatment and control groups was 3.85% (one case of small blisters on the skin) and 7.69% (one case of small blisters on the skin and one case of a cutaneious infection); however, the difference was not statistically significant (*P* > 0.05) [37]. Another study reported the incidence of adverse reactions in the treatment and control groups was 8.89% (one case of rash, one case of headache, and two cases of pruritus) and 6.67% (one case of rash, one case of headache, and one case of pruritus); the difference was not statistically significant (*P* > 0.05) [32]. No drug-related severe liver and kidney adverse events occurred, suggesting that GQD had fewer adverse events during the treatment and safety was acceptable.

### 3.4. Publication Bias

**Figure 8:**
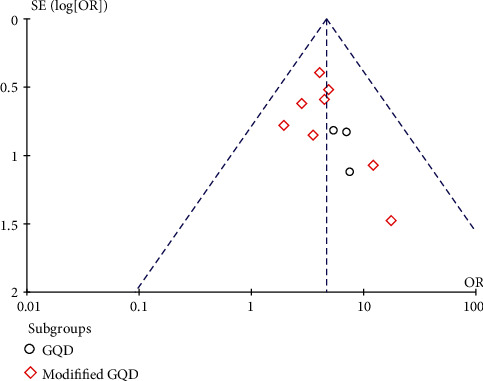
Funnel plot of the effective rate.

A funnel plot was drawn for the effective rate of the 11 RCTs. The incomplete symmetry shown in the funnel plot suggested that there was publication bias. We speculate that the publication bias was related to the incomplete literature search and the differences in the efficiency criteria among the included RCTs (Figure 8).

### 3.5. Sensitivity Analysis

The subgroups with an *I*^2^ > 50% in the forest plot of the duration of diarrhea, fever, and vomiting, and the time to correct dehydration were all subjected to sensitivity analysis to determine the source of heterogeneity by the article-by-article exclusion method. The results showed that *I*^2^ was still >50%, suggesting that the results were relatively robust.

## 4. Discussion

### 4.1. Interpretations

Our study evaluated the effectiveness and safety of GQD in the treatment of children with diarrhea. Review Manager 5.4 software was used to analyze the clinical data of 11 RCTs, involving 1126 participants. All of the trials were carried out in China. The results of the meta-analysis suggest that the GQD treatment group had significant differences with respect to shortening the duration of diarrhea, fever, vomiting, and correcting dehydration compared with the control group, indicating that GQD may have advantages in improving the clinical effectiveness. At the same time, accompanied by fewer adverse reactions and satisfactory safety.

### 4.2. Strengths

Several issues that were revealed during the meta-analysis are worthy of attention. First, in determining the duration of fever and vomiting heterogeneity, we showed that regional difference was an influencing factor. Children who reside in the southern region of China had a longer duration of fever and vomiting due to high temperatures and humidity, which is consistent with previous research findings [40]. Therefore, a multicenter/multiregion RCT should be planned to avoid regional differences affecting the results [41]. In this way, the regional differences can be taken into consideration during the specific analysis, because regional differences include many factors that affect the occurrence of diseases, such as climate, economy, ethnicity, sanitary conditions, and living habits [42]. Second, it was surprising that GQD had better efficiency than modified GQD. A previous study counted 250 prescriptions used by Zhang Zhongjing, and reported that the average number of herbal medicines in his prescriptions was 4.61, such streamlined prescriptions had a better curative effect than complicated prescriptions [43]. The researchers of RCTs seem to have discovered this phenomenon, so the prescriptions used in RCTs in recent years were all unmodified GQD (Figure 7). GQD is derived from the classic TCM prescriptions that was written by Zhang Zhongjing in the Han Dynasty. GQD consists of only 4 herbs (Radix *Puerariae lobatae* (Ge Gen), Radix *Scutellariae* (Huang Qin), *Rhizoma coptidis* (Huang Lian), and Radix *Glycyrrhizae* (Gan Cao)). GQD is traditionally and clinically used to treat both the “external and internal symptoms” of diarrhea with fever [44] and is known for its streamlined prescription. Third, in determining the duration of diarrhea heterogeneity, we have showed that the course of treatment was an influencing factor. We also showed that GQD played a more significant role when the course of treatment was ≥5 days. Previous studies have shown that GQD has a significant inhibitory effect on pathogenic bacteria, such as *Staphylococcus aureus* and *Escherichia coli* in the intestinal tract [45]. Based on this finding we speculate that the curative effect is affected by the treatment course and may be related to the metabolic cycle of the intestinal flora, and the adjustment of the intestinal flora is not apparent within a treatment course of <5 days [46]. It has been reported that GQD regulates the intestinal flora and increases the abundance of beneficial bacteria that can produce short-chain fatty acids (SCFAs) [24]. The increased levels of SCFAs could help attenuate mucosal proinflammatory responses by inhibiting histone deacetylase and the NF-KB pathway [24]. It has been reported that diarrhea-related deaths and episodes are mainly attributed to rotavirus and intestinal bacteria [5]. Through the network pharmacology analysis, it has been concluded that there are 130 active ingredients in GQD that is used in the treatment rotavirus enteritis, including flavonoids, alkaloids, phenyl esters, and fatty acids [47]. The main flavonoids have positive effects on antioxidative stress and immune regulation [48]. In addition, flavonoids have an antibacterial effect [49]. Based on this, we found that GQD as a treatment prescription is robust.

### 4.3. Limitations

There were some potential limitations to our study that need to be addressed in the future. First, in recent years, traditional Chinese herbal medicines have been gradually recognized by international medicine, but classic prescriptions, such as GQD, are only active in China, the trials carried out are also limited to China, and the patients who benefit from the herbal medicines are also limited to Chinese patients. Therefore, most of the RCTs related to GQD are also published in Chinese journals, which leads to the lack of international recognition of the therapeutic effect of GQD. Second, because TCM emphasizes “individual treatment” and “treatment based on syndrome differentiation,” the dosage, course of treatment, and method of administration between RCTs are also different. These factors may cause high heterogeneity. Third, as shown in Table 1 and Figure 1, many RCTs that were included lacked demographic information and detailed descriptions of trial blinding methods. These factors might have affected the analysis quality of this study.

## 5. Conclusion

However, despite the above-mentioned limitations, this study confirmed that the therapeutic effect and safety of GQD as a TCM for the treatment of pediatric diarrhea cannot be ignored. In addition, this study concluded the following: (1) it is recommended that GQD be used to treat pediatric diarrhea for >5 days. (2) Multicenter RCTs should be conducted to avoid the impact of regional differences on the results. (3) Unmodified GQD is recommended because it may have better efficacy.

## Figures and Tables

**Table 1:** Characteristics of the included RCTs.

RCT	Sample size	Age (mean or range)	Course of the disease (mean or range)	Intervention		Outcome	Region	Cause of disease
	E/C	E/C	E/C	E	C			
Sha et al. [22]	60/60	1.53/1.56 y	1.72/1.69 d	GQD + A	B + A	①②⑥	South	Others
Xu et al. [21]	40/38	(12.56 ± 0.58)/(12.86 ± 0.6)m	—	GQD + B	B	②⑥⑧	South	Others
Liu [35]	96/68	—	—	GQD	C	①②④⑥	South	Others
Ma et al. [33]	46/46	(1.82 ± 0.61)/(1.96 ± 0.54)y	(1.61 ± 0.59)/(1.69 ± 0.65)d	GQD + B + A	B + A	①②③⑥⑦⑩⑪	North	Virus
Shi et al. [32]	45/45	(2.02 ± 0.67)/(2.05 ± 0.69)y	(6.86 ± 1.57)/(6.88 ± 1.61)d	GQD + D + B + A	D + B + A	①②③④⑤⑥⑦⑫	North	Rotavirus
Lu et al. [34]	192/192	(25.32 ± 6.32)/(24.28 ± 5.55)m	(2.12 ± 1.05)/(2.34 ± 1.11)m	GQD + B + A	B + A	①②⑥	South	Others
Zhang and Li [31]	54/50	(1.9 ± 0.4)/(1.7 ± 0.3)y	(1.1 ± 0.3)/(0.9 ± 0.5)d	GQD + E + C + A	E + C + A	①②③④⑥⑨	South	Others
Huang et al. [38]	30/30	(2.18 ± 0.25)/(2.25 ± 0.26)y	(2.28 ± 0.26)/(2.21 ± 0.25)d	GQD + C + B + A	C + B + A	①②③⑤⑥⑦	South	Rotavirus
Huang and Dong [37]	26/26	(1.71 ± 0.24)/(1.73 ± 0.25)y	(7.34 ± 1.02)/(7.09 ± 0.98)d	GQD + F + A	F + A	①②③④⑥⑫	South	Others
Fang and Hang [39]	29/22	(11.54 ± 2.68)/(11.57 ± 2.64)m	(3.41 ± 0.22)/(3.38 ± 0.20)d	GQD + C + B + A	C + B + A	①②③④⑥⑬	South	Virus
Li and Jiang [36]	72/72	(1.72 ± 0.89)/(1.82 ± 0.75)y	(2.14 ± 1.05)/(2.33 ± 1.12)d	GQD + A	A	①②③④⑥	South	Rotavirus

Abbreviations: E = experimental group; C = control group; d = days; y = years; m = months; A = conventional treatment (rehydration infusion, antifever, and correction of water and electrolyte disorders); B = montmorillonite powder (smecta); C = ribavirin; D = bifidobacterium; E = racecadotril; F = acupoint application; ①: duration of fever; ②: duration of diarrhea; ③: duration of vomiting; ④: duration of correcting dehydration; ⑤: duration of virus becoming negative; ⑥: effective rate; ⑦: symptoms of traditional Chinese medicine; ⑧: intestinal lactose level; ⑨: serum inflammatory factors; ⑩: intestinal flora; ⑪: barrier function of intestinal mucosa; ⑫: serum myocardial enzyme spectrum; ⑬: immune function index; “—” = the specific data are not shown, but it has been noted that the difference is not significant (*P* > 0.05) in trials.

**Table 2:** Ingredients of GQD used in the 11 RCTs.

RCT	TCM	The way of taking medicine	Course of treatment (day)	Ingredients	Adverse event
Sha et al. [22]	Modified GQD (1 dose/d)	Oral	3	*Puerariae lobatae* radix (Ge Gen) 10 g, *scutellariae* radix (Huang Qin) 6 g, *Coptidis rhizoma* (Huang Lian) 3 g, *Glycyrrhizae* radix (Gan Cao) 3 g, *Atractylodis rhizoma* (Cang Zhu) 6 g, *Pogostemonis herba* (Huo Xiang) 6 g, magnoliae officinalis cortex (Hou Po) 6 g, citri reticulatae pericarpium (Chen Pi) 6 g, poria (Fu Ling) 10 g, and mume fructus (Wu Mei) 3 g	NM

Xu et al. [21]	Modified GQD (2 ml/kg, tid-qid)	Oral	5	*Puerariae lobatae* radix (Ge Gen), *Scutellariae* radix (Huang Qin), *Coptidis rhizoma* (Huang Lian), *Glycyrrhizae* radix (Gan Cao), *atractylodis rhizoma* (Cang Zhu), poria (Fu Ling), hordei fructus germinatus (Mai Ya), setariae fructus germinatus (Gu Ya), raphani semen (Lai Fuzi), *Atractylodis macrocephalae* rhizoma (Bai Zhu), and galli gigerii endothelium corneum (Ji Neijin)	NM

Liu [35]	Modified GQD (1 dose/d)	Oral	—	*Puerariae lobatae* radix (Ge Gen) 5 g, *Scutellariae* radix (Huang Qin) 4 g, *Coptidis rhizoma* (Huang Lian) 3 g, pogostemonis herba (Huo Xiang) 6 g, *Atractylodis macrocephalae* rhizoma (Bai Zhu) 6 g, amomi fructus rotundus (Bai Doukou) 3 g, *Isatudis* radix (Ban Langen) 12 g, indigo naturalis (Qing Dai) 3 g, papaveris pericarpium (Ying Suke) 2 g, *Aucklandiae* radix (Mu Xiang) 3 g, mume fructus (Wu Mei) 12 g, and poria (Fu Ling) 10 g	NM

Ma et al. [33]	Modified GQD (2 ml/kg, bid)	Oral	7	*Puerariae lobatae* radix (Ge Gen) 8 g, *Scutellariae* radix (Huang Qin) 6 g, *Coptidis rhizoma* (Huang Lian) 5 g, *Glycyrrhizae* radix (Gan Cao) 3 g, *Alismatis rhizoma* (Ze Xie) 6 g, *Aucklandiae* radix (Mu Xiang) 8 g, and poria (Fu Ling) 8 g	NM

Shi et al. [32]	GQD (1 dose/d)	Oral	3–6	*Puerariae lobatae* radix (Ge Gen) 9 g, *Scutellariae radix* (Huang Qin) 6 g, *Coptidis rhizoma* (Huang Lian) 6 g, and *Glycyrrhizae* radix (Gan Cao) 3 g	M

Lu et al. [34]	Modified GQD (1 dose/d)	Oral	3	*Puerariae lobatae* radix (Ge Gen) 10 g, *Scutellariae* radix (Huang Qin) 6 g, *Coptidis rhizoma* (Huang Lian) 3 g, G*glycyrrhizae* radix (Gan Cao) 3 g, poria (Fu Ling)10 g, *Atractylodis rhizoma* (Cang Zhu) 6 g, pogostemonis herba (Huo Xiang) 6 g, magnoliae officinalis cortex (Hou Po) 6 g, citri reticulatae pericarpium (Chen Pi) 6 g, and mume fructus (Wu Mei) 3 g	NAE

Zhang and Li [31]	Modified GQD (1 dose/d)	Oral	3	*Puerariae lobatae* radix (Ge Gen) 10 g, *Scutellariae* radix (Huang Qin) 6 g, *Coptidis rhizoma* (Huang Lian) 3 g, *Glycyrrhizae radix* (Gan Cao) 3 g, poria (Fu Ling) 10 g, pogostemonis herba (Huo Xiang) 6 g, magnoliae officinalis cortex (Hou Po) 6 g, mume Fructus (Wu Mei) 3 g, *Atractylodis* rhizoma (Cang Zhu) 6 g, and citri reticulatae pericarpium (Chen Pi) 6 g	NAE

Huang et al. [38]	GQD (1 dose/d)	Oral	3–7	*Puerariae lobatae* radix (Ge Gen) 9 g, *Scutellariae* radix (Huang Qin) 6 g, *Coptidis rhizoma* (Huang Lian) 6 g, and *Glycyrrhizae* radix (Gan Cao) 3 g	NM

Huang and Dong [37]	GQD (1 dose/d)	Oral	7	*Puerariae lobatae* radix (Ge Gen) 15 g, *sScutellariae* radix (Huang Qin) 9 g, *Coptidis rhizoma* (Huang Lian) 3 g, and *Glycyrrhizae* radix (Gan Cao) 5 g	M

Fang and Hang [39]	Modified GQD (1 dose/d)	Oral	3	*Puerariae lobatae* radix (Ge Gen) 15 g, *Scutellariae* radix (Huang Qin) 10 g, *Coptidis rhizoma* (Huang Lian) 6 g, *Glycyrrhizae* radix (Gan Cao) 6 g, plantaginis semen (Che Qianzi) 12 g, massa medicata fermentata (Shen Qu) 10 g, and *Aucklandiae* radix (Mu Xiang) 6 g	NAE

Li and Jiang [36]	Modified GQD (1 dose/d)	Oral	—	*Puerariae lobatae* radix (Ge Gen) 10 g, *Scutellariae* radix (Huang Qin) 5 g, *Coptidis rhizoma* (Huang Lian) 5 g, *Glycyrrhizae* radix (Gan Cao) 5 g, and *Atractylodis macrocephalae* rhizoma (Bai Zhu) 10 g	NAE

Abbreviations: “—” = not explicitly mentioned in the trials; NM = not mentioned; M = mentioned; NAE = no adverse events.

## Data Availability

The data supporting this meta-analysis were obtained from previously reported studies and datasets, which have been cited in this article.

## Supplementary Materials

Supplementary Materials Table S1: search strategy. Table S2: PRISMA checklist.
