# Supplementary material for: Efficacy and Safety of Gegen Qinlian Decoction for Pediatric Diarrhea: A Systematic Review and Meta-Analysis
Source: Evid Based Complement Alternat Med. 2022 Sep 8;2022:4887259. doi: 10.1155/2022/4887259 (PMC9477636; doi:10.1155/2022/4887259)
Supplement: Supplementary Materials — Table S1: search strategy. Table S2: PRISMA checklist. [file 4887259.f1.zip › Table S2 Search Strategy.pdf]

Databases	Retrieval strategy
CNKI<br>(Chinese)	#1 SU=（“小儿腹泻”+“儿科腹泻”+“小儿肠炎”+“儿科肠炎”）<br>#2 SU=（“葛根芩连汤”）<br>#3 FT=（“随机”+“盲法”+“安慰剂”）<br>#4 #1 AND #2 AND #3
VIP<br>(Chinese)	#1 题名或关键词：小儿腹泻+儿科腹泻+小儿肠炎+儿科肠炎<br>#2 题名或关键词：葛根芩连汤<br>#3 #1 AND #2
WanFang<br>(Chinese)	#1主题：“小儿腹泻”+主题：“儿科腹泻”+主题：“小儿肠炎”+主题：“儿科肠炎”<br>#2主题：“葛根芩连汤”<br>#3全部：“随机”+全部：“盲法”+全部：“安慰剂”<br>#4 #1 AND #2 AND #3
CBM<br>(Chinese)	#1 “小儿腹泻”[加权：扩展]<br>#2 “小儿腹泻”[常用字段：智能]OR“儿科腹泻”[常用字段：智能]<br>#3 “小儿肠炎”[常用字段：智能]OR“儿科肠炎”[常用字段：智能]<br>#4 （#1）OR（#2）OR（#3）<br>#5 “葛根芩连汤”[常用字段：智能]<br>#6 （#4）AND（#5）<br>#7 “随机”[全部字段：智能]OR“盲法”[全部字段：智能]OR “安慰剂”[全部字段：智能]
	#1 “pediatric diarrhea” [MeSH Terms]<br>#2 “children's diarrhea” [Title/Abstract]<br>#3 “diarrhea in children” [Title/Abstract]<br>#4 “infantile diarrhea” [Title/Abstract]<br>#5 “children's enteritis” [Title/Abstract]<br>#6 “enteritis in children” [Title/Abstract]

PubMed	#7 “pediatric enteritis” [Title/Abstract]]<br>#8 (#1 OR #2 OR #3 OR #4 OR #5 OR #6 OR #7)<br>#9 “Gegen Qinlian decoction” [Title/Abstract]<br>#10 “Gegen Qinlian” [Title/Abstract]<br>#11 “Ge Gen Qin Lian” [Title/Abstract]<br>#12 (#9 OR #10 OR #11)<br>#13 “randomized controlled trial” [Publication Type] OR “randomized” [Title/Abstract] OR “placebo” [Title/Abstract]<br>#14 (#8 AND #12 AND #13)
